# High monoamine oxidase a expression predicts poor prognosis for prostate cancer patients

**DOI:** 10.1186/s12894-023-01285-8

**Published:** 2023-07-04

**Authors:** Lingxiao Chen, Wei Xiong, Lin Qi, Wei He

**Affiliations:** Department of Urology, Xiangya Hospital, Central South University, No. 87 Xiangya Road, Changsha, Hunan Province 410008 People’s Republic of China

**Keywords:** MAOA, Prostate cancer, Prognosis, Immunohistochemistry, Radical prostatectomy

## Abstract

**Background:**

Monoamine oxidase A (MAOA) is a mitochondrial enzyme that is involved in prostate tumorigenesis and cancer metastasis. The predictive power of the preoperative clinical and pathological indicators for prostate cancer(PC) remains to be improved. To enrich evidence regarding the value of MAOA as a prognostic biomarker in clinical practice, this study explored the significance of MAOA expression as a prognostic marker for patients with PC after radical prostatectomy-pelvic lymph node dissection (RP-PLND).

**Methods:**

MAOA expression was analyzed in 50 benign prostate tissues and 115 low-intermediate risk and 163 high-risk PC tissues using tissue immunohistochemistry (IHC). Propensity score matching, survival analysis and COX regression analysis were conducted to investigate the correlation between high MAOA expression and progression free survival (PFS) in PC patients.

**Results:**

MAOA expression was increased in PC patients, especially in those with high risk PC and pathological lymph node (pLN) metastasis. High MAOA expression was significantly associated with PSA recurrence for both low-intermediate risk PC patients (log-rank test: *P =* 0.02) and high risk PC patients (log-rank test: *P =* 0.03). Cox regression analysis revealed that high MAOA expression was an adverse prognostic factor for both low-intermediate risk PC patients (hazard ratio [HR] 2.74, 95% confidence interval [CI] 1.26–5.92; *P =* 0.011) and high risk PC patients (HR 1.73, 95% CI 1.11–2.71; *P =* 0.016). High MAOA expression was also significantly associated with PSA recurrence in high risk PC patients developed into castration-resistant prostate cancer (CRPC) and were receiving abiraterone therapy (log-rank: *P =* 0.01).

**Conclusions:**

MAOA expression correlates with the malignant progression of PC. High MAOA expression may be a poor prognostic marker for patients with PC after RP-PLND. More careful follow up or potential of adjuvant hormonal therapy may be addressed for patients with high MAOA expression.

**Supplementary Information:**

The online version contains supplementary material available at 10.1186/s12894-023-01285-8.

## Introduction

Prostate cancer (PC) is the most common genitourinary malignancy and the second leading cause of male cancer death in the United States [1, 2]. The popularization of PSA screening has contributed to a significant increase in the diagnosis of PC along with patients undergoing radical prostatectomy-pelvic lymph node dissection (RP-PLND) in the past few years [3]. One of the challenges in PC treatment is whether all patients who undergo surgery would need active adjuvant therapy. Currently, there are very few biomolecular markers available for evaluating the malignant progression of PC after RP-PLND. For a long period, serum prostate specific antigen (PSA), Gleason score (GS) and clinical stage have been shown to be important predictive indices of prognosis [4–6]. However, for those PCs in the intermediate range of a given index, the predictive power of the preoperative clinical and pathological indicators is limited. With routing genetic testing and molecular characterization of patients with either metastatic castration-resistant prostate cancer (mCRPC) or locally advanced PC being a feasible approach to select patients who are more likely to respond to targeted agents, minimizing toxicities from unnecessary therapies and tailoring a more efficient patient-based treatment, especially biomarkers development for precision, tailored medicine in PC management been accelerated by liquid biopsy, more and more potential biomolecular markers available for evaluating the malignant progression of PC after RP-PLND need to be further clinically testified [7, 8]. In this study, a propensity scoring-based retrospective study was carried out to evaluate a new molecular marker to better predict the prognosis for PC patients and achieve better treatment outcome after radical surgery.

Monoamine oxidase A (MAOA) is a mitochondrial enzyme which catalyzes the degradation of monoamine neurotransmitters such as 5-hydroxytryptamine (5-HT) [9, 10]. The process produces a major source of reactive oxygen species (ROS) thus hydrogen peroxide (H_2_O_2_) which can cause epithelial-mesenchymal transition (EMT) by stabilizing HIF1αand lead to tumor initiation and progression by damaging DNA of cancer cells [11]. A growing body of evidence indicates that MAOA either has altered expression levels or exerts a regulatory effect in a variety of types of cancer [12, 13]. In PC, several studies revealed a potential role for MAOA in mediating prostate tumorigenesis and cancer metastasis [14, 15]. These findings suggest MAOA could be a potential prognostic molecule for PC. However, the research of MAOA in PC prognosis is relatively lacking and the value of MAOA as a predictive biomarker in clinical practice remains to be explored.

In this study, to evaluate the clinical relationship between MAOA and PC, MAOA expression detection in the prostate tissues of PC patients combined with a survival analysis and Cox regression analysis after propensity score matching was accomplished.

## Materials and methods

### Prostate specimens and patients follow up

In this study, two hundred and seventy-eight PC tissues, and 50 benign prostate biopsies were collected during January 2012 to January 2016 in our department. The PC tissues were collected from PC patients going through RP-PLND and all the operative margins were negative. Benign prostate biopsies were collected from patients going through prostate biopsies with no malignancy diagnosed by pathologists. The risk stratification was according to the criteria from Mayo Clinic came up by professor D’Amico AV [16]. Low-intermediate risk PC was defined as: PSA ≤ 20 ng/mL, pT < T_2c_ and GS < 8. High-risk PC was defined as: PSA > 20 ng/mL, pT ≥ T_2c_ or GS ≥ 8 (Table S). PC patients with pLN metastatic were all from high-risk PC patients. All authors had access to information that could identify individual participants during or after data collection.

Tumor staging and grading was performed as the same guidelines as previous [17]. No patient went into neoadjuvant endocrine-therapy. Patients with high risk PC(PSA > 20 ng/mL, pT ≥ T_2c_ or GS ≥ 8) or pLN metastasis were recommended for endocrine therapy after RP-PLND [18]. The average follow-up period of the patients after RP-PLND was 3 years. Patients with high risk PC who developed CRPC were recommended with abiraterone therapy and followed up for 18 months. Progression-free survival (PFS) was defined as the period of time when no PSA recurrence was detected in living patients after RP-PLND. The local ethic committee reviewed and approved the study protocol. All patients provided written informed consent for tissue samples. All the patients signed an informed consent form. The study was approved and consented by the Xiangya hospital ethics committee.

### Tissue immunohistochemistry (IHC)

IHC analysis of 278 primary PC and 50 benign prostate samples was performed using antibodies against MAOA (1:200, H-70, Santa Cruz) following our published protocol with minor modifications and the scoring classification [19]. Positive reactions were defined as those showing brown signals in the cell cytoplasm. For MAOA, a staining index (values, 0–12) was determined by multiplying the score for staining intensity with the score for positive area. The intensity was scored as follows: 0, negative; 1, weak; 2, moderate; and 3, strong. The frequency of positive cells was defined as follows: 0, less than 5%; 1, 5% to 25%; 2, 26% to 50%; 3, 51% to 75%; and 4, greater than 75%. When the staining was heterogeneous, it was scored as follows: each component was scored independently and summed for the results. For example, a specimen containing 75% tumor cells with moderate intensity (3 × 2 = 6) and another 25% tumor cells with weak intensity (1 × 1 = 1) received a final score of 6 + 1 = 7. For statistical analysis, scores of 0 to 7 were considered low expression and scores of 8 to 12 considered high expression. MAOA immunoreactivity was scored by two independent pathologists who lacked prior knowledge of the patients’ clinicopathological characteristics. In cases of discrepant results, the values were discussed until an agreement was reached. Each sample was observed in 3 different areas with the highest score chosen for the final score.

### Statistical analysis

Multiple clinical and pathologic characteristics of patients with PC, including PC features (PSA, GS), were collected as previous described [19]. As differences existed in the above-mentioned patient and tumor characteristics among the cohort groups with low MAOA expression and high MAOA expression, 1:1 propensity score matching was conducted between the cohort groups in low-intermediate risk and high-risk PC patients. Propensity score matching balanced the differences of characteristics including Age, ECOG PS, Pathological stage, PSA,and GS in the cohorts.

Comparisons of above-mentioned patient and tumor characteristics were conducted using the chi-squared test or unpaired *t*-test. PFS in the cohort groups was examined using Kaplan–Meier and log-rank tests. To determine the prognostic variables, the association of follow up outcomes with high MAOA expression and the above-mentioned patient and tumor characteristics was evaluated using Cox regression analysis after propensity score matching. All statistical analyses were conducted using the IBM SPSS Statistics 24.

## Results

### High MAOA expression is associated with high risk PC and pLN metastasis

**Fig. 1:**
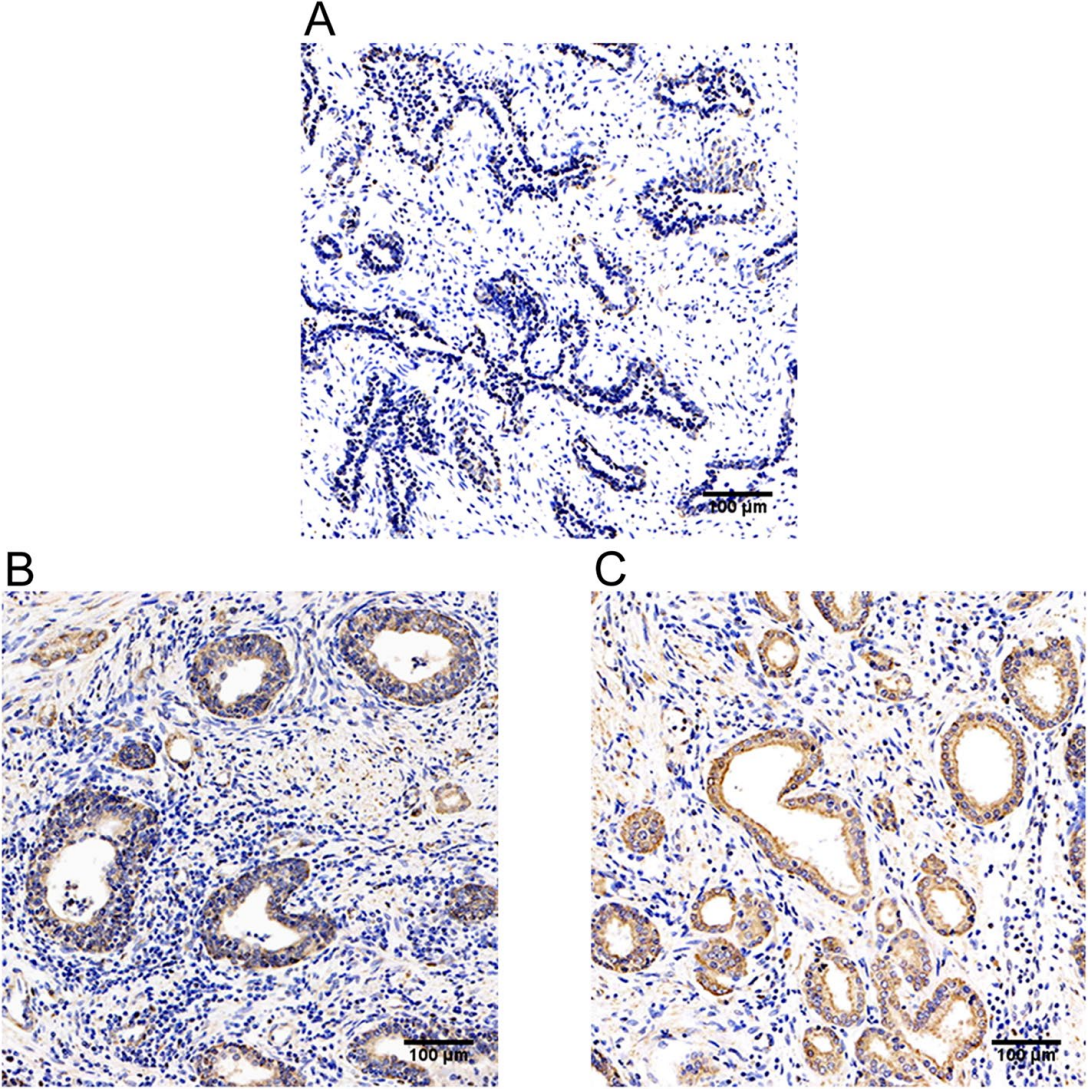
MAOA immunohistochemistry. (A) Low MAOA expression in benign prostate tissue (magnification × 200). (B) High MAOA expression in a high risk PC (magnification × 200). (C) High MAOA expression in PC with pLN metastasis (magnification × 200)

Of the 278 PC patients with an average age of 64 years (range 50 to 73 years), 115 patients were diagnosed with low-to-intermediate risk PC and 163 patients were diagnosed as high risk PC. All the patients went through RP-PLND. Twenty-eight patients with high risk PC were found to have pLN metastasis after RP-PLND. MAOA expression was mainly found in the cytoplasm by IHC (Fig. 1). Observation of MAOA expression revealed that the lowest MAOA expression was observed in benign prostate tissues with only 10 (20%) specimens having high MAOA expression, and MAOA expression was higher in PC tissues with 46 of 115 (40%) low-intermediate risk PC specimens and 75 of 135 (55.6%) high risk PC specimens with high MAOA expression (Table 1). The highest MAOA expression was observed in PC tissues from patients with pLN metastasis in which 22 of 28 (78.6%) specimens had high MAOA expression (Table 1).

**Table 1:** Comparison of MAOA expression between benign prostate tissues and PC. (L: low expression; H: high expression; ^a^, ^b^, ^c^, ^d^: group symbols)

	Total	MAOA-L	MAOA-H	χ2	*P*
	*N =* 328	cases (%)	cases (%)		
**Mean MAOA scores**	**4(2 ~ 7)**	**10(8 ~ 12)**			
Benign prostate	50	40(80.0%)	10(20.0%)		
tissues^a^					
Low-intermediate	115	69(60.0%)	46(40.0%)	6.58	**0.010**
risk^b^					
High risk^c^	135	60(44.4%)	75(55.6%)	6.00	**0.014**
pLN metastatic PC^d^	28	6(21.4%)	22(78.6%)	5.46	**0.019**

Meanwhile, high MAOA expression was found to be associated with the risk factors of high PSA and high GS (Table 2). This outcome implied that MAOA expression increases with prostate tumorigenesis and metastasis. MAOA may be a biological molecule involving in the progression of PC.

**Table 2:** Comparison of MAOA expression. (L: low expression; H: high expression)

Total	MAOA-L	MAOA-H	χ2	*P*
*N =* 278	cases (%)	cases (%)		
Pathological stage				
< T_2b_	44(32.6%)	30 (21.0%)		
T_2b_	45(33.3%)	52 (36.4%)		
≥ T_2c_	46(34.1%)	61(42.6%)	5.05	**0.08**
PSA (ng/mL)				
≤ 10	46(34.1%)	22(15.4%)		
10–20	52(38.5%)	49(34.3%)		
> 20	37(27.4%)	72(50.3%)	19.96	** < 0.01**
GS				
< 7	50(37.0%)	19(13.3%)		
7	42(31.1%)	45(31.5%)		
> 7	43(31.9%)	79 (55.2%)	25.10	** < 0.01**

### High MAOA expression is correlated with biochemical recurrence of PC patients after RP-PLND

The characteristics of the low-intermediate risk PC patients are summarized in Table 3. There were 69 low-intermediate risk patients with low MAOA expression and 46 low-intermediate risk patients with high MAOA expression in the cohorts before propensity score matching. Significant differences were observed in the PSA and GS between the two groups. After propensity score matching, 42 patients in each group were matched and included as new cohorts with no differences in PSA and GS.

**Table 3:** Clinical information of low-intermediate risk PC patients with low or high MAOA expression after RP-PLND. (L: low expression; H: high expression)

	MAOA-L	MAOA-H	*P*	MAOA-L	MAOA-H	*P*
	cases (%)	cases (%)		cases (%)	cases (%)	
Age **(years)**	50–71	53–71	0.13	50–71	53–71	0.98
	(60.5 ± 10.5)	(62 ± 9)		(60.5 ± 10.5)	(62 ± 9)	
ECOG PS ≥ 1						
No	49(71.0%)	25(54.3%)		28(66.7%)	25(59.5%)	
Yes	20(29.0%)	21(45.7%)	0.077	14(33.3%)	17(40.5%)	0.65
Pathological stage						
< T_2b_	26(37.7%)	14(30.4%)	14(33.3%)	14(33.3%)		
T_2b_	43(62.3%)	32(69.6%)	0.55	28(66.7%)	28(66.7%)	1
PSA (ng/mL)						
≤ 10	32(46.4%)	12(26.1%)		14(33.3%)	12(28.6%)	
> 10	37(53.6%)	34(73.9%)	**0.033**	28(66.7%)	30(71.4%)	0.81
GS						
< 7	34(49.3%)	13(28.3%)		11( 26.2%)	11(26.2%)	
7	35(50.7%)	33(71.7%)	**0.033**	31(73.8%)	31(73.8%)	1

The characteristics of high risk PC patients are summarized in Table 4. There were 66 patients with low MAOA expression and 97 with high MAOA expression in the cohorts before propensity score matching. Significant differences existed in PSA, GS and pLN metastasis between the two groups. After propensity score matching, 58 patients in each group were matched and included as new cohorts with no differences in PSA, GS and pLN metastasis.

**Table 4:** Clinical information of high risk PC patients with low or high MAOA expression after RP-PLND. (L: low expression; H: high expression)

	MAOA-L	MAOA-H	*P*	MAOA-L	MAOA-H	*P*
	cases (%)	cases (%)		cases (%)	cases (%)	
Age (years)	53–73	52–71	0.60	53–73	52–71	0.51
(60.0 ± 13.0)	(60.5 ± 9.5)			(63.0 ± 10.0)	(62 ± 9)	
ECOG PS ≥ 1						
No	36(54.5%)	64(66.0%)		32(55.2%)	34(58.6%)	
Yes	30(45.5%)	33(34.0%)	0.19	26(44.8%)	24(41.4%)	0.85
Pathological stage						
< T_2c_	20(30.3%)	36(37.1%)		20(34.5%)	22(37.9%)	
≥ T_2c_	46(69.7%)	61(62.9%)	0.40	38(65.5%)	36(62.1%)	0.85
PSA (ng/mL)						
≤ 20	29(43.9%)	25(25.8%)		21(36.2%)	17(29.3%)	
> 20	37(56.1%)	72(74.2%)	**0.018**	37(63.8%)	41(70.7%)	0.55
GS						
< 8	23(34.8%)	18(18.6%)		18(31.0%)	14(24.1%)	
≥ 8	43(65.2%)	79(81.4%)	**0.027**	40(69.0%)	44(75.9%)	0.53
pLN						
0	60(90.1%)	75(77.3%)		52(89.7%)	49(84.5%)	
1	6(9.9%)	22(22.7%)	**0.033**	6(10.3%)	9(15.5%)	0.58

**Fig. 2:**
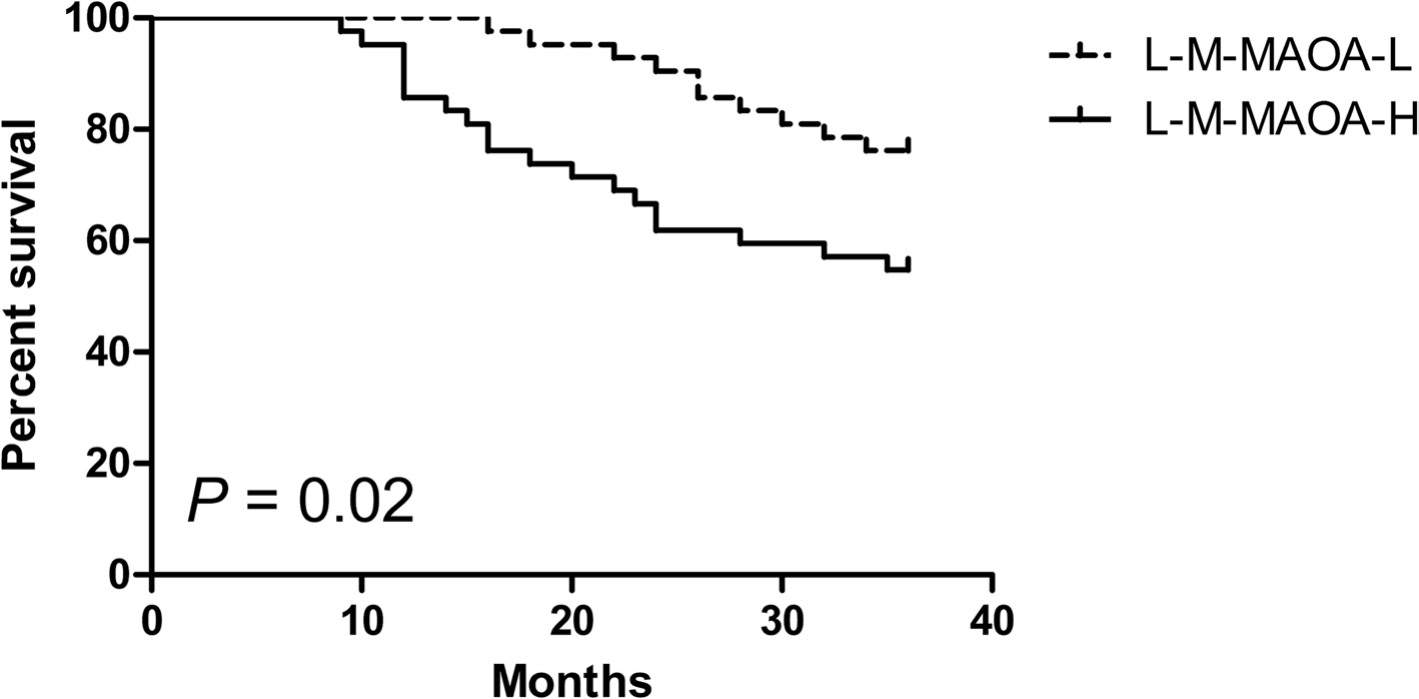
Kaplan–Meier estimated PFS curves for low-intermediate risk PC patients with low MAOA expression compared with the PFS curves for patients with high MAOA expression. (L-M: low-intermediate risk prostate cancer; MAOA-L: low MAOA expression; MAOA-H: high MAOA expression)

**Fig. 3:**
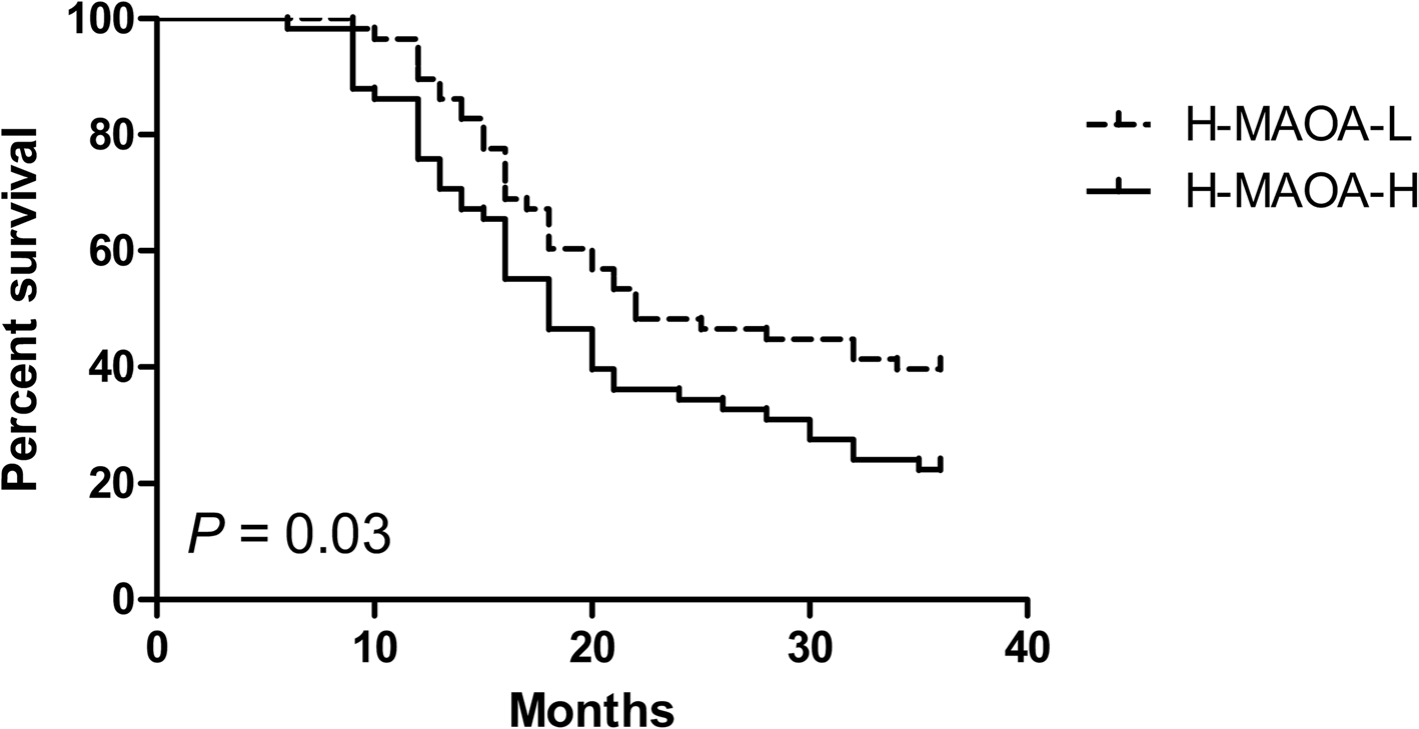
Kaplan–Meier estimated PFS curves for high risk PC patients with low MAOA expression compared with the PFS curves for patients with high MAOA expression. (H: high risk prostate cancer; MAOA-L: low MAOA expression; MAOA-H: high MAOA expression)

Kaplan–Meier analysis indicated that high MAOA expression is correlated with poor PFS not only in low-intermediate risk PC patients but also in high-risk PC patients. Among the patients with low-intermediate risk PC, the low-MAOA-expression group had a 2-year PFS rate of 90.5% versus 61.9% of 2-year PFS rate in the high-MAOA-expression group (log-rank:* P =* 0.02) (Fig. 2). The median event-free survival time of the low-MAOA-expression group was 2.5 years versus 2 years in high-MAOA-expression group. Among the patients with high-risk PC, the low-MAOA-expression group had a 2-year PFS rate of 48.3% versus 34.5% of 2-year PFS rate in the high-MAOA-expression group (log-rank:* P =* 0.03) (Fig. 3). The median event-free survival time of the low-MAOA-expression group was 1.8 years versus 1.5 years in high-MAOA-expression group.

Univariate Cox regression analysis excluded insignificant factors of age and ECOG both in groups of low-intermediate risk PC patients and high risk PC patients. Multivariate Cox regression analysis showed that high MAOA expression (hazard ratio [HR] 2.74, 95% confidence interval [CI] 1.26–5.92; *P =* 0.011), pT = T_2b_ (HR 5.46, 95% CI 1.88–15.90; *P* < 0.01), PSA > 10 ng/mL (HR 5.58, 95% CI 1.68–18.53; *P* < 0.01) and GS = 7 (HR 6.55, 95% CI 1.55–27.64; *P =* 0.011) were independent predictors of worse PFS in low-intermediate risk PC patients (Table 5). Furthermore, high MAOA expression (HR 1.73, 95% CI 1.11–2.71; *P =* 0.016), pT ≥ T_2c_ (HR 2.42, 95% CI 1.41–4.17; *P* < 0.01), PSA > 20 ng/mL (HR 2.01, 95% CI 1.16–3.47; *P =* 0.012), GS ≥ 8 (HR 2.60, 95% CI 1.43–4.73; *P* < 0.01) and pLN positivity (HR 5.73, 95% CI 2.69–12.20; *P* < 0.01) were independent predictors of poor PFS in high risk PC patients (Table 6).

**Table 5:** Propensity score-adjusted univariate and multivariate Cox regression analysis for factors affecting PFS in 42 low-intermediate risk patients. (H: high expression)

	Univariate analysis			Multivariate analysis		
	*P*	HR	95% CI	*P*	HR	95% CI
Age (≥ 65)	0.87	0.94	0.45–1.96			
ECOG PS ≥ 1	1.00	1.00	0.47–2.11			
pT = T_2b_	**0.014**	3.79	1.32–10.89	** < 0.01**	5.46	1.88–15.90
GS = 7	**0.017**	5.75	1.37–24.21	**0.011**	6.55	1.55–27.64
PSA > 10	**0.011**	4.70	1.42–15.53	** < 0.01**	5.58	1.68–18.53
MAOA-H	**0.025**	2.41	1.12–5.18	**0.011**	2.74	1.26–5.92

**Table 6:** Propensity score-adjusted univariate and multivariate Cox regression analysis for factors affecting PFS in 58 high risk patients. (H: high expression)

	Univariate analysis			Multivariate analysis		
	*P*	HR	95% CI	*P*	HR	95% CI
Age (≥ 65)	0.25	0.77	0.50–1.20			
ECOG PS ≥ 1	0.15	1.38	0.89–2.14			
pT ≥ T_2c_	** < 0.01**	2.51	1.51–4.18	** < 0.01**	2.42	1.41–4.17
GS ≥ 8	** < 0.01**	2.11	1.22–3.65	** < 0.01**	2.60	1.43–4.73
PSA > 20	** < 0.01**	2.08	1.25–3.46	**0.012**	2.01	1.16–3.47
pLN +	** < 0.01**	11.95	5.86–24.35	** < 0.01**	5.73	2.69–12.20
MAOA-H	**0.038**	1.60	1.03–2.49	**0.016**	1.73	1.11–2.71

**Fig. 4:**
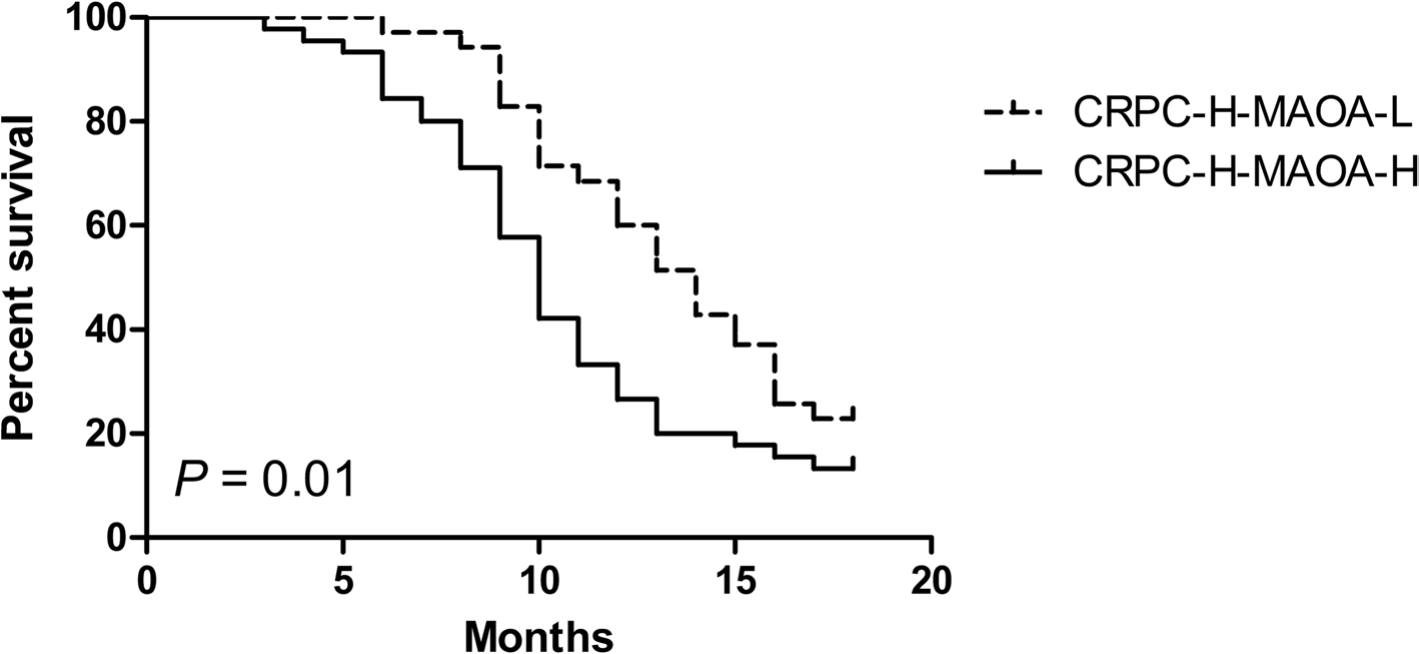
Kaplan–Meier estimated PFS curves for high risk PC patients developing into CRPC and receiving abiraterone therapy with low MAOA expression compared with the PFS curves for patients with high MAOA expression. (CRPC: castration resistant prostate cancer; H: high risk; MAOA-L: low MAOA expression; MAOA-H: high MAOA expression)

Furthermore, Kaplan–Meier analysis indicated that high MAOA expression was correlated with poor PFS in 58 high risk PC patients developing into CRPC and receiving abiraterone therapy, the low-MAOA-expression group had a 1-year PFS rate of 60% versus a 1-year PFS rate of 26.7% in the high-MAOA-expression group (log-rank: *P =* 0.01) (Fig. 4). The median event-free survival time of the low-MAOA-expression group was 1 year versus 0.8 year in high-MAOA-expression group.

## Discussion

Tumor stage, PSA and GS have been the most important predictive parameters for the follow-up of patients with PC, which plays a core role in clinical practice [16]. To most extensively improve the outcome of patients with PC after radical surgery, more meaningful and valuable biological markers are needed to further stratify the patients for more accurate and positive surveillance. MAOA is a mitochondrial membranebound enzyme that catalyzes the degradation of biogenic and dietary monoamines by oxidative deamination [20]. One of MAOA’s key functions is to induce EMT by producing excessive intracellular levels of hydrogen peroxide, a major reactive oxygen species by-product generated by MAOA-mediated enzymatic reactions, in which cancer cell aggressiveness, invasiveness, and metastatic potential are increased [9]. Studies have shown a potential association between increased MAOA expression, PC progression, and worse clinical outcomes [9, 21]. However, there is limited evidence regarding the value of MAOA as a prognostic biomarker in clinical practice. This study focuses on the role of MAOA as a prognostic biomarker in predicting prognosis of PC patients in clinic.

The relationship between MAOA and PC progression and prognosis was studied to evaluate the role of MAOA as a predictive biomarker for patients with PC. Our investigation revealed that MAOA expression increased with PC progression. High MAOA expression was found in high-risk PC patients, especially those with pLN metastasis. Moreover, high MAOA expression was found to be significantly associated with higher tumor stage, GS, and PSA, Which was consistent with the previous study implying MAOA expression was significantly elevated in Gleason 4 or 5 samples relative to Gleason 3 samples [21]. Based on the above outcomes, it was suggested that MAOA could be a biological molecule marking PC aggressiveness and a determinant factor determining patient prognosis. Further survival analysis following propensity score-based matching explored the efficacy of MAOA expression in predicting PSA recurrence for patients with PC after RP-PLND. The unbalanced distribution of PSA, GS, and pLN metastasis was balanced by propensity score matching between the cohort groups in low-intermediate-risk PC patients and high-risk PC patients. Kaplan–Meier analysis demonstrated that high MAOA expression was associated with PSA recurrence and was verified as a risk factor by the calculated HR in the multivariate Cox regression analysis. Forward follow-up of high-risk patients with PC developing CRPC revealed a correlation between high MAOA expression and poor prognosis in patients receiving abiraterone therapy. These outcomes largely were in accordance with rough report that MAOA-low patients had significantly enhanced survival times when compared with MAOA-high patients by previous research [9]. These data confirms a close relationship between high MAOA expression and prognosis for patients with PC.

Considering these findings, MAOA may act as a risk stratification indicator for the clinical assessment of patients with PC. For patient prognosis, low MAOA expression may be beneficial, whereas high MAOA expression may be disadvantageous.

The novelty of this study lies in that the propensity score matching maximumly balanced biases embedded in some critical clinical characteristics of the cohorts potentially affecting accurate evaluation of MAOA expression’s influence on prognosis. The limitations of the current study were the retrospective nature of the analysis. Only an ideally designed prospective and randomized study can definitively validate these findings. Nevertheless, the results of this study imply tremendous underlying promise for MAOA in the clinical evaluation of PC treatment and prognosis. For example, low or middle-risk PC patients might be further risk stratified by MAOA expression regarding to specific surgery option, neoadjuvant therapy, follow up and prognosis to improve patient outcomes.

## Conclusions

In conclusion, high MAOA expression represents an increased risk for PC patients. High MAOA expression was significantly correlated with prognosis for PC patients concerning biochemical recurrence after RP-PLND. The identification of MAOA as a predictive marker could improve risk stratification and guide personalized treatment approaches for PC patients.

## Supplementary Information

**Additional file 1:** **Table S.** Features of PC patients assessing low and high risk.

## Data Availability

The datasets generated and analysed during the current study are not publicly available due to patients’ privacy but are available from the corresponding author on reasonable request.
