# Supplementary material for: High monoamine oxidase a expression predicts poor prognosis for prostate cancer patients
Source: BMC Urol. 2023 Jul 4;23:112. doi: 10.1186/s12894-023-01285-8 (PMC10318740; doi:10.1186/s12894-023-01285-8)
Supplement: Supplementary file 1 — Additional file 1: Table S. Features of PC patients assessing low and high risk. [file 12894_2023_1285_MOESM1_ESM.pdf]

Table S. Features of PC patients assessing low and high risk.

Low risk	Middle risk	High risk
$pT_{1\sim2a}$	pT_{2b} or	pT_{2c} or
PSA < 10ng/mL	PSA 10-20 ng/mL or	PSA > 20 ng/mL or
GS < 7	GS 7	GS > 7
		$pT_{3\sim4}$ or pLN+ (any PSA, any GS)
